# Dexmedetomidine Ameliorate CLP-Induced Rat Intestinal Injury via Inhibition of Inflammation

**DOI:** 10.1155/2015/918361

**Published:** 2015-07-26

**Authors:** Yanqing Chen, Liyan Miao, Yusheng Yao, Weilan Wu, Xiaodan Wu, Cansheng Gong, Liangcheng Qiu, Jianping Chen

**Affiliations:** Department of Anesthesiology, Fujian Provincial Hospital and The Shengli Medical Clinical College of Fujian Medical University, Fuzhou 350001, China

## Abstract

The aim was to verify that dexmedetomidine (DEX) can attenuate CLP-induced intestinal injury via inhibition of inflammation. Male Sprague-Dawley (SD) rats were randomly allocated into Sham group and the other three CLP model groups, in terms of different treatments: placebo, DEX, and yohimbine plus DEX (DEX + YOH) groups. Pathology examination was conducted with HE stain. To identify differences among groups, the levels of DAO, and D-lactate in serum were measured by spectrophotometry, and the levels of TNF-*α*, IL-1*β*, and IL-6 in serum and organ were measured by ELISA. The expressions of occludin and TLR4 in tissue were detected by Western blot. The survival rate of an additional group of animals within 7 d was recorded. In DEX group, mortality was lower, histology change was minor, DAO, and D-lactate levels were reduced, and occludin expression was increased; the expressions of TNF-*α*, IL-1*β*, IL-6, and TLR4 were also decreased in DEX group. These results indicated that acute intestinal injury induced by CLP was mitigated by DEX treatment. However, these effects of DEX were significantly attenuated by yohimbine in DEX + YOH group. Our study indicated the protective effects of DEX on CLP-induced injury, which may be associated with the inhibition of inflammation via modulating TLR4 pathway and can be blocked by yohimbine.

## 1. Introduction

Sepsis, characterized by hyperinflammatory state, was complex and devastating, which can lead to catastrophic effects. Despite the fact that progressions were made in diagnosis and treatment of these patients, the morbidity and mortality of rates of sepsis remained constantly high, up to 50% [1–3]. Excessive synthesis of proinflammatory cytokine induced by severe sepsis causes damage to vital organs, including intestine [4, 5].

Among the immune system in human body, intestinal mucosa served as key protective barrier, of which the dysfunction with accompanying bacterial translocation can be fatal to sepsis patients for the associated systemic inflammatory response and consequent impairments of the end-organ function [6, 7]. Such sequential reactions were considered to be crucial in etiology of sepsis induced multiorgan dysfunction syndrome (MODS) [8–10]. Since intestine plays a key role in the pathogenesis of sepsis, the method for the treatment of intestinal damage emerged endlessly, including reasonable use of antibiotics [11] or low-dose corticosteroids [12], nutritional support therapy [13], and regulation of intestinal flora [14].

Dexmedetomidine (DEX) has been regarded as a highly selective *α*_2_-adrenoceptor agonist, which mostly applied to different clinical settings for sedative or analgesic requirements [15]. Its attenuation of inflammation by activation on the *α*_2_-adrenoceptors was confirmed by several investigations [16, 17]. Clinical evidence suggested that DEX sedated with DEX will suppress the inflammatory response mediated by TNF-*α*, IL-1*β*, IL-6, and so on, for septic patients [18]. Animal studies also revealed that the inhibitory efficacy of DEX on released cytokine in inflammatory responses and subsequently decreased in mortality [19, 20]. The anti-inflammatory properties of DEX by exerting protective effects on different organs and tissues have been noted by several experiments [21–23]. However, whether the administration of DEX can affect the sepsis induced intestinal injury remains unclear. On the basis of the characteristics of DEX from previous studies, we designed this research to verify the hypothesis that DEX can ameliorate CLP-induced intestinal injury via inhibition of inflammation.

## 2. Materials and Methods

### 2.1. Animals

Adult male Sprague-Dawley (SD) rats (220~270 g) were obtained from Laboratorial Animal Center of Fujian Medical University. All animals were kept in standard environment with temperature and humidity control under a 12 h day/night cycle and free access to food and drink condition. The study protocols were approved by the institutional review board (IRB) and conducted in compliance with the institutional criteria for the practical care and use of laboratory animals in biomedical research.

### 2.2. CLP Model [24]

All rats were anesthetized with intraperitoneal injection of 10% chloral hydrate (0.3 mL/100 g) before surgical procedures, while their body temperatures were maintained at 36~38°C by a heating pad. An intravenous 24-gauge catheter was installed in tail vein for drug and saline administration. Next, after skin shaving and the preparation of abdominal wall with 10% povidone-iodine solution, a 2 cm incision was then performed through midline. The cecum was carefully isolated and ligated at distal to the ileocecal valve with a 4-0 silk suture to avoid intestinal obstruction and then punctured twice with a sterile 20-gauge needle and gently squeezed to extrude a small amount of feces from the perforation sites. We tried to guarantee the quantity of extruded feces was consistent in all rats. The quantity of extruded feces were limited (small droplet, about 1 mm in diameter) and consistent in all rats. Then placed back the cecum, the abdominal cavity was closed in two layers with continuous suture of 3-0 silk. The group Sham was treated in an identical manner, but no cecal ligation or puncture was performed. Each rat received a subcutaneous injection of 1 ml normal saline for fluid resuscitation after surgery. All animals were well tolerated throughout the procedures without further sedatives to maintain immobility, and no antibiotics were administrated during then.

### 2.3. Experimental Protocol and Drug Administration

A total of 64 rats were randomly assigned into four groups (*n* = 16 per group): the Sham group, cecum ligation and puncture (CLP) group, CLP plus intravenous injection of DEX (5 *μ*g·kg^−1^·h^−1^, Hengrui Medicine, Jiangsu, China) 30 minutes after CLP for 1 h (DEX) group, and combination of *α*_2_-adrenergic antagonist yohimbine (1.0 mg/kg, injection process lasting no less than 15 min, Sigma Chemical Co., St. Louis, MO, USA) prior to DEX treatment (DEX + YOH) group. The infusion rate was fixed at 5 mL·kg^−1^·h^−1^ [25] to eliminate the influences of fluid load; therefore the concentrations of drugs were different individually. The rats in Sham and CLP groups received normal saline over the same period. The doses of DEX and yohimbine used in the present study were according to the previous research [20, 26, 27].

### 2.4. Mortality Rate Observation

The survival studies were conducted on 10 animals from each group as the above. The mortality and 7-day survival rate after surgery were investigated through close follow-up; the estimated probability was analyzed by Kaplan-Meier survival curve.

### 2.5. Serum and Intestinal Tissues Collection

At 12 h and 24 h after CLP, blood samples were harvested from the inferior vena cava (3 mL of each); the serum was separated by centrifugation at 3000 rpm for 15 min at 4°C and then stored at −80°C for ELISA and spectrophotometry analysis. Then the rats were sacrificed for a 0.5~1.0 cm segment of the terminal ileum 5 cm proximal to the ileocecal valve, fixed in 4% formaldehyde for pathological examination. Two segments of intestine (300 mg) from 10 cm to ileocecal valve were obtained and immediately frozen in liquid nitrogen; then the intestinal tissues were homogenized and centrifuged at 12,000 rpm at 4°C for 15 minutes; the supernatant was collected for Western blot and ELISA.

### 2.6. Pathological Examination of Intestine

The tissues of intestine were embedded in paraffin wax and sectioned at 5 *μ*m and then stained with hematoxylin and eosin (HE) and examined under a microscope (Nikon, Tokyo, Japan). The Pathological changes were evaluated by the same pathologist, who was blinded to the experimental protocols.

### 2.7. Determination of the Levels of Diamine Oxidase (DAO) and D-Lactate by Spectrophotometric Assay and TNF-*α*, IL-1*β*, and IL-6 by ELISA

The levels of DAO and D-lactate in serum were measured using spectrophotometry [28] and enzyme-linked ultraviolet spectrophotometry [29], respectively (Sigma Chemical Co., St. Louis, MO, USA). TNF-*α*, L-1*β*, and IL-6 in serum and intestine were measured by commercial ELISA kits (Bio Swamp Life Science Inc., Wuhan, China) in accordance with the manufacturers' instructions.

### 2.8. Determination the Expressions of TLR4 and Occludin by Western Blot

The protein concentration in the supernatant fluid was measured by BCA protein assay. Equal amounts of protein were subjected to SDS-PAGE and transferred to PVDF membranes. The transferred proteins were blocked with 5% skim milk and then incubated overnight with anti-TLR4, anti-occludin, and anti-*β*-actin primary antibodies (Cell Signaling Technology Inc., NY, USA), respectively. Immunoreactivity was detected with horseradish peroxidase conjugated secondary antibodies (Sigma Chemical Co., St. Louis, MO, USA) and visualized by enhanced chemiluminescence detection film.

### 2.9. Statistical Analysis

The normality of distribution was assessed with the Kolmogorov–Smirnov test. Parametric data were reported as mean (standard deviation (SD)) and nonparametric data were reported as median and interquartile range (IQR). SPSS 20.0 software was used for experimental results analysis. DAO, D-lactate, TNF-*α*, IL-1*β*, IL-6, and TLR4 among groups were analyzed using one-way analysis of variance (ANOVA). Post hoc comparisons were made with least significant difference (LSD) test. Intergroup differences between two time points were analyzed with the unpaired Student's* t*-test. The survival analyses among groups were compared using Kaplan–Meier and the Mantel–Cox methods. Statistical significance was defined as *P* < 0.05.

## 3. Results

### 3.1. Mortality Rate

**Figure 1:**
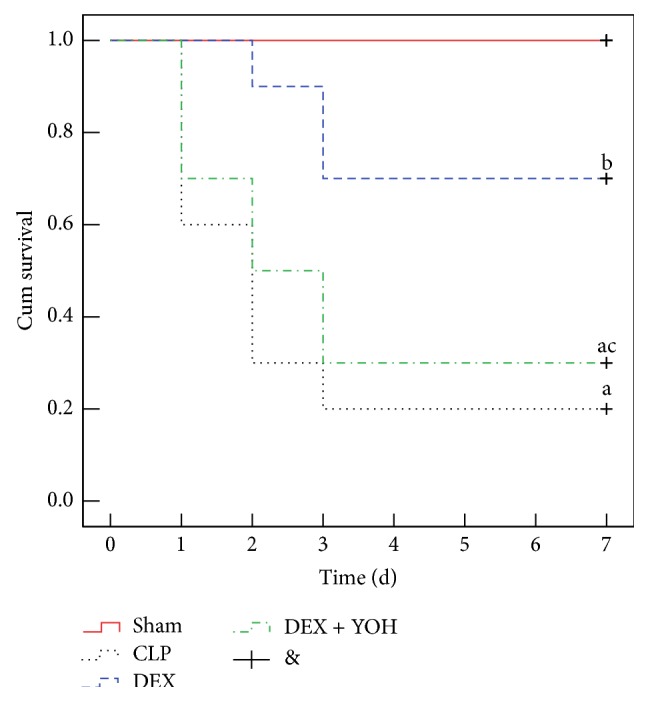
DEX treatment improved the survival of CLP-induced septic rats. Kaplan–Meier survival curves for four groups of rats 7 d (*n* = 10). ^a^ *P* < 0.05, versus Sham group; ^b^ *P* < 0.05, versus CLP group; ^c^ *P* < 0.05, versus DEX group.

As shown in Figure 1, the mortality rates within 7 d in Sham, CLP, DEX, and DEX + YOH groups were 0%, 80%, 30%, 70%, respectively. Most of the deaths occur within the first 3 days. The mortality rate was higher in CLP group and DEX + YOH group than in Sham group (*P* < 0.05) but lower in DEX group than in CLP and DEX + YOH group (all *P* < 0.05). No significant diffidence was found between CLP and DEX + YOH groups (*P* > 0.05).

### 3.2. DEX Alleviated Morphology of Intestine Injury Induced by Sepsis

**Figure 2:**
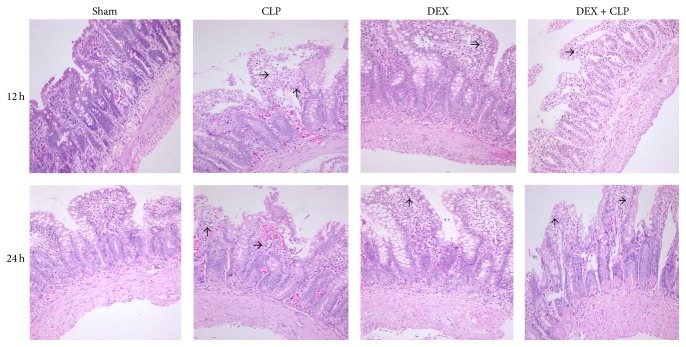
DEX treatment alleviated intestinal injury induced by CLP. Microscopic findings of the intestines stained with HE at 12 h and 24 h in Sham, CLP, DEX, and DEX + YOH groups (×200).

The pathological analysis showed integrated intestinal mucosa and compactly arrayed epithelium in the rats of Sham group (Figure 2). However, glands of the intestines were significantly damaged; severe edema of mucosal villi and neutrophil infiltration were observed in the rats of CLP and DEX + YOH groups at 12 h and 24 h after the induction of sepsis. By contrast, DEX ameliorated the pathological injury of the ileum with improved villi height and reduced neutrophil infiltration compared with sepsis group.

### 3.3. Detection of the Levels of DAO and D-Lactate in Serum and the Expression of Occludin in Intestine

**Figure 3:**
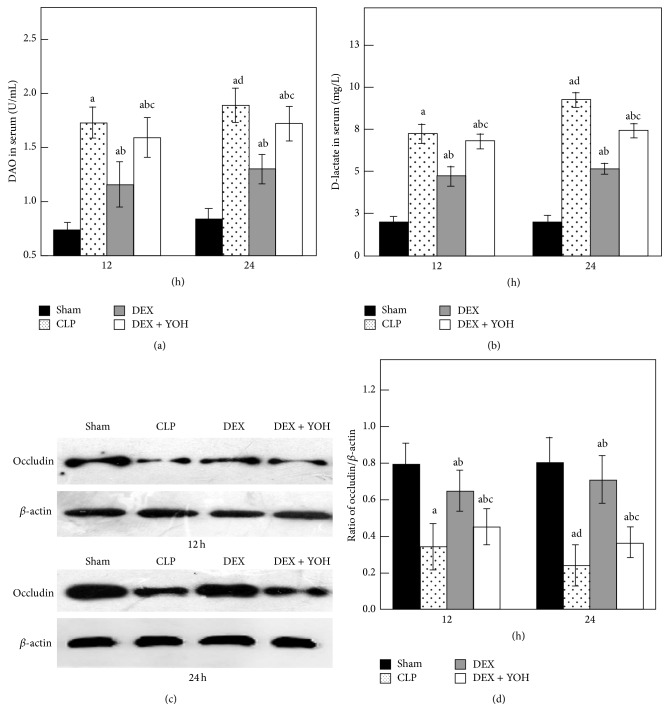
DAO and D-lactate in serum and occludin in intestine in experiment rats among four groups at 12 h and 24 h (*n* = 8). Data are expressed as mean ± SD. ^a^ *P* < 0.05, versus Sham group; ^b^ *P* < 0.05, versus CLP group; ^c^ *P* < 0.05, versus DEX group; ^d^ *P* < 0.05, versus CLP group 12 h.

Pathological examination is relatively crude to evaluate organ injury. Therefore, biochemical tests were performed to evaluate intestine damage. A similar pattern of changes was noted in intestinal barrier function (Figure 3). The serum DAO was significantly increased at 12 h and 24 h in CLP group compared with Sham group (*P* < 0.05) and more so in 24 h than 12 h following the operation. Treatment with DEX 5 *μ*g·kg^−1^·h^−1^ resulted in lower DAO levels at 12 h and 24 h, respectively, compared to CLP group (*P* < 0.05). A more specific marker of intestinal barrier function, D-lactate, was also measured at 12 h and 24 h after CLP. The D-lactate levels were consistent with the DAO levels and showed a similar trend among the groups indicating that DEX can alleviate intestinal permeability in the CLP model. Occludin plays a key role in maintaining the integrity of intestinal barrier. Our results show that the level of the occludin was reduced in intestine of CLP rats compared with Sham controls (*P* < 0.05) and was more serious in 24 h. Taken together, the data presented in Figure 3 indicate that obvious intestinal injury occurred in septic mice. In contrast, DEX restored the inhibition of occludin expression (*P* < 0.05). The effects of DEX were partially inhibited by yohimbine (*P* < 0.05) relative to CLP rats.

### 3.4. Effect of DEX on the Levels of TNF-*α*, IL-1*β*, and IL-6 in Serum and Intestine

**Figure 4:**
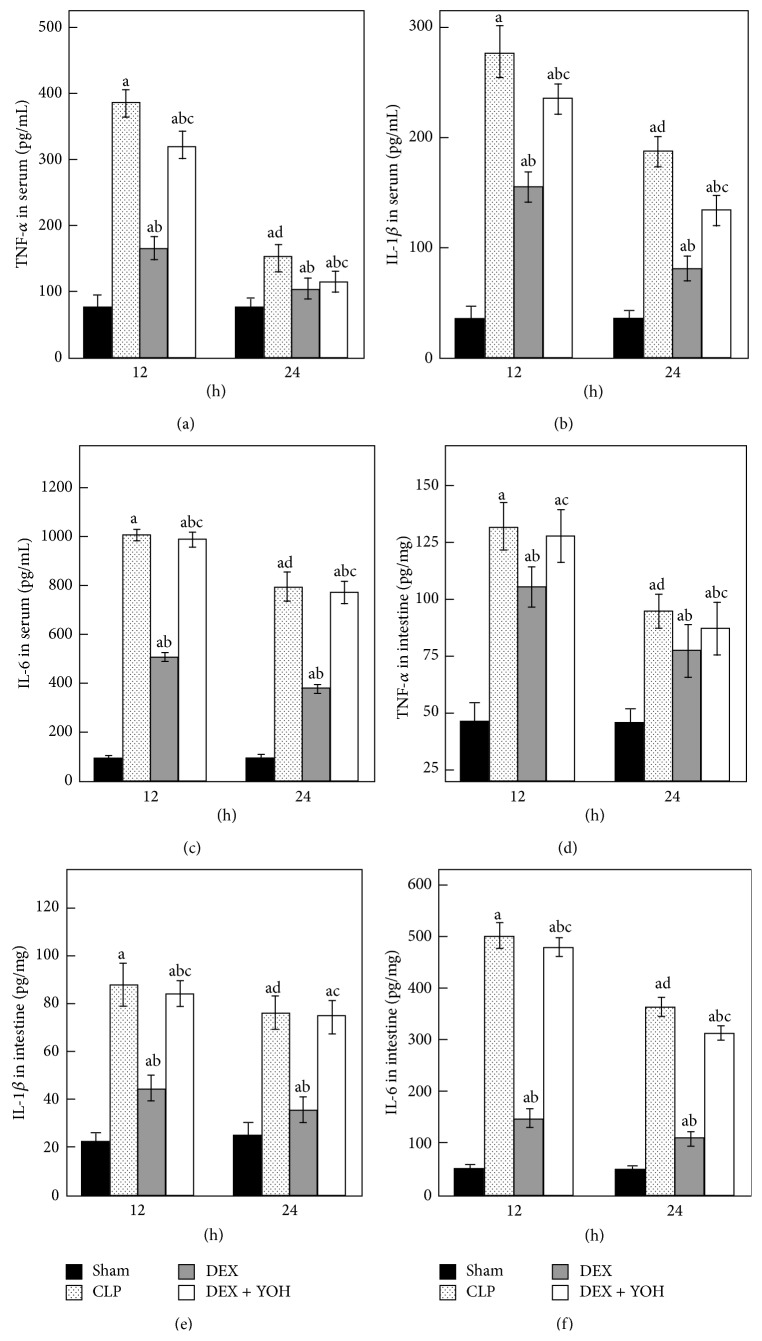
TNF-*α*, IL-1*β*, and IL-6 in experiment rats among four groups (*n* = 8). Data are expressed as mean ± SD. ^a^ *P* < 0.05, versus Sham group; ^b^ *P* < 0.05, versus CLP group; ^c^ *P* < 0.05, versus DEX group; ^d^ *P* < 0.05, versus CLP group 12 h.

As shown in Figure 4, the levels of TNF-*α*, IL-1*β*, and IL-6 in serum were significantly higher in the CLP group than in the Sham group (all *P* < 0.05). In addition to activity of inflammatory factors in serum, expressions of TNF-*α*, IL-1*β*, and IL-6 in intestine were also all increased in CLP group compared to Sham group. The levels of inflammatory factors were significantly decreased at 24 h but were still higher than normal. DEX significantly inhibited the rise of TNF-*α*, IL-1*β*, and IL-6 (all *P* < 0.05) compared to CLP rats, which were effectively reversed by cotreatment with yohimbine (*P* < 0.05, versus DEX group), respectively. There was no significant difference between CLP group and DEX + YOH group in terms of intestinal TNF-*α* at 12 h and IL-1*β* at 24 h (*P* > 0.05).

### 3.5. Effect of DEX on the Expression of TLR4 in Intestine

**Figure 5:**
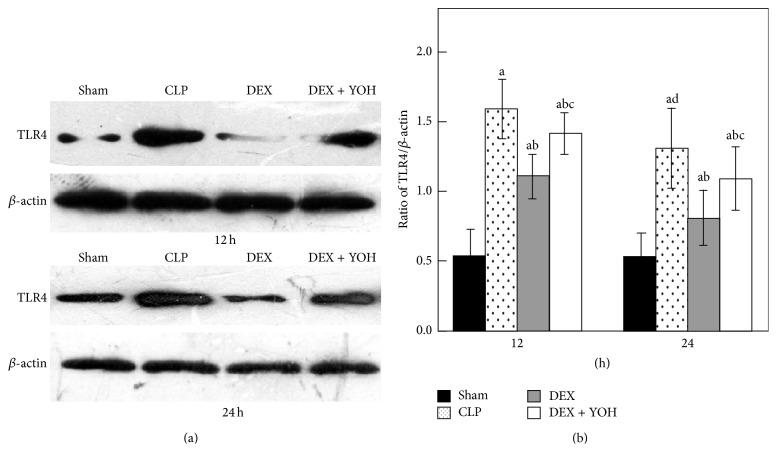
Expression of TLR4 in intestine by Western blotting in experiment rats among four groups (*n* = 8). Data are expressed as mean ± SD. ^a^ *P* < 0.05, versus Sham group; ^b^ *P* < 0.05, versus CLP group; ^c^ *P* < 0.05, versus DEX group; ^d^ *P* < 0.05, versus CLP group 12 h.

To investigate the molecular mechanisms of DEX induced anti-inflammatory effect, we assessed TLR4 expression in intestine using Western blotting (Figure 5) in 12 h and 24 h. There was a weak expression of TLR4 in the Sham group. However, untreated septic rats significantly increased the expression of TLR4 compared with Sham group (*P* < 0.05). The higher levels of TLR4 in septic rats were observed at 12 h. In contrast, septic rats treated with DEX exhibited a decrease in the expression of TLR4 compared with untreated septic rats (*P* < 0.05). Yohimbine reduced but could not completely abolish the effect of DEX, suggesting that anti-inflammatory effect may be partially dependent on *α*_2_-adrenoceptors.

## 4. Discussion

Experimental sepsis in our study was induced with the cecal ligation and puncture (CLP) technique to establish standard animal model of sepsis in mice [30]. Considering the high mortality rate in rats and the elevated concentrations of inflammatory factors in serum, it was indicated that the model used in our study was successful. In this present study, the application of DEX alleviated the pathological damage in intestine tissue, significantly attenuated in concentrations of DAO and D-lactate, and increased the expression of occludin. Besides, DEX also decreased the expression levels of TNF-*α*, IL-1*β*, IL-6, and TLR4 in intestine. These results indicated that DEX can ameliorate damage to intestine tissue by modulating inflammatory responses, which could be associated with the inhibition of TLR4 receptor in CLP-induced sepsis.

Investigations on the influence of gastrointestinal barrier injury in severe sepsis and subsequent MODS have increased over time [10]. Changes of histology and permeability in intestinal mucosa correlated with the cytokines and intestinal bacteria-endotoxin translocation [31]. The pathogenesis has not yet been fully understood; however, prompt and effective treatment with antibiotics affects the prognosis in clinical settings. Substantial evidences provided by previous studies prove that DEX has beneficial effects in sepsis condition. Lai et al. demonstrated that DEX suppressed the overexpression of inflammatory mediators in LPS activated murine macrophages [32]. Sezer et al. found that DEX may have protective effect on liver pathological changes in sepsis [33]. Shi et al. showed that DEX can protect against the lung tissue injury of LPS-induced sepsis in rats [34]. These evidences suggested that DEX can alleviate acute organ injuries during sepsis, which is consistent with our study that DEX can greatly impact the CLP-induced sepsis that resulted in a significant decrease in mortality.

As a functional intestinal marker enzyme, concentrations of DAO in plasma increased with the intestine inflammation, integrity of intestine mucosal, or permeability of the intestinal barrier [35]. D-lactate is one of the metabolites of luminal microbiota, whose level indicated the severity of intestine injury and also consistently changed with the epithelium function of the intestinal barrier [36]. In our study, septic rats revealed serious pathological damage including the destruction of intestinal mucosa and inflammatory cell infiltration. Elevated DAO and D-lactate in serum indicated that intestinal mucosal mechanical barrier was severely damaged in CLP model. The present results demonstrated that administration of DEX had beneficial effects on maintaining the integrity of intestinal mucosa barrier, as indicated by the reduction of DAO and D-lactate. In addition, DEX suppressed the reduction of tight junction protein occludin, which is the structural basis of mechanical barrier [37]. The decrease in occludin directly reflected the damage of intestinal mucosa induced by CLP. DEX also improved the intestines histological examination. The levels of DAO and D-lactate among the groups were consistent with the pathological evaluation. Therefore, these results indicate that DEX may avoid intestinal injury during sepsis.

It was known that an increase in the levels of inflammatory cytokines, including TNF-*α*, IL-1*β*, and IL-6, plays important role in the process of intestinal injury [4]. TNF-*α* is widely accepted as the initial mediators in the pathogenesis of sepsis; it plays a key role in endogenous inflammation to induce IL-1*β* and IL-6 production during the early phase of CLP. In the current study, DEX reduced TNF-*α*, IL-1*β*, and IL-6 production in serum and intestine, which was partially in accordance with previous study of the inhibitory effects of DEX on cytokines [38].

A growing body of evidence demonstrates that Toll-like receptors (TLRs) are highly conserved protein receptors involved in the pathogenesis of critical illnesses, including sepsis. TLR4, together with CD14 and MD2 adapter molecule, has gained the most attention and was reported playing a pivotal role in the initiation and development of immune responses to microbial pathogens [39, 40]. It has been shown to be triggered by damage-associated molecular patterns (DAMPs). DAMPs bind to TLR4 leading to the activation of downstream signaling molecules including NF-*κ*B and MAPKs. These could augment the production of proinflammatory cytokines (TNF-*α*, IL-1*β*, IL-6, etc.) and eventually lead to robust inflammatory response [41]. LPS, TNF-*α*, or IL-1*β* can also trigger the generation of TLR4, which means that TLR4 may participate in “cross talk” for the amplification of inflammatory responses [42]. To further explore the mechanism of DEX protective effect on intestine, TLR4 was examined. Our data are consistent with the hypothesis that level of TLR4 was elevated in septic rats but was significantly attenuated by DEX. These data provide strong evidence that inhibition of the TLR4 may contribute to anti-inflammatory effect in sepsis.

Collectively, these results indicated that the protective effects of DEX on septic intestinal injury might be partially achieved by altering the expressions of TLR4. In fact, our results are partly consistent with a previous study which demonstrated that DEX suppressed the TLR4 mediated inflammatory circuitry [32, 43].

In addition, yohimbine, a nonspecific *α*_2_-adrenoceptor antagonist, was applied in the current study to further explore whether *α*_2_-adrenoceptors are involved in the protective effect of DEX. It is believed that DEX provided its effect via *α*_2_-adrenoceptors, since yohimbine partly reversed the protective effect of DEX because of the disappearance of its anti-inflammatory function. It has been previously reported that yohimbine itself does not suppress inflammation [27, 44]. Besides that, based on the result of previous research [7], we concluded that yohimbine abolished the intestinal protective effect of dexmedetomidine but it had no effect on intestinal injury during intestinal ischemia-reperfusion in rats. The results indicated that the therapeutic effects of DEX might be mediated, at least in part, by *α*_2_-adrenoceptors during sepsis. Nevertheless, dexmedetomidine can also activate I_1_-type imidazoline receptors [45]; previous studies indicated that imidazoline receptors possess significant anti-inflammatory capacity [46, 47]. So it is possible that the aforementioned effect of DEX is related to its effects on imidazoline receptor.

Our current research chose a single dose of DEX (5 *μ*g·kg^−1^·h^−1^), which was based on previous findings. It seems to be relatively high compared with the clinical doses used to produce anesthesia in humans. The recommended safe and effective clinical dosage range of DEX is only 0.5–1 *μ*g/kg [48]. However, recent papers indicate that some patients might need higher doses of DEX (maybe 2−5 times higher than the usual clinical dose) to achieve adequate sedation [49]. Simultaneously, there may be differences in the doses of DEX between different species. Further investigation is needed on this point.

Several possible limitations of the present study should be noted. First, we demonstrated the inhibition effect of DEX on proinflammatory mediators, but it still is unclear whether DEX has any effect on the production of anti-inflammatory cytokines such as IL-10. Second, we did not maintain plasma levels of DEX, but intravenous injection usually results in good plasma levels. We do not monitor the hemodynamics in rats, so it is difficult to distinguish whether the protective effect is affected by hemodynamics fluctuations. Finally, we only studied short-time effects of DEX on CLP induced injury in intestine but more doses and long-term effect of DEX also need to be clarified.

## 5. Conclusion

In conclusion, our study indicated that DEX could exert protective role against sepsis-induced intestinal barrier dysfunction via attenuating intestinal inflammatory responses and then effectively prevent the development of sepsis. Thus, our results provide a new insight for the clinical use of DEX and suggest that DEX sedation may have a therapeutic potential in reducing intestinal injury associated with sepsis.
